# Chinese herb ultrafine powder supplementation improves egg nutritional value and quality in laying hens

**DOI:** 10.1080/01652176.2024.2331530

**Published:** 2024-04-01

**Authors:** Jue Gui, Md. Abul Kalam Azad, Wenchao Lin, Chengwen Meng, Xin Hu, Yadong Cui, Wei Lan, Jianhua He, Xiangfeng Kong

**Affiliations:** a Key Laboratory of Agro-ecological Processes in Subtropical Region, Hunan Provincial Key Laboratory of Animal Nutrition Physiology and Metabolic Processes, National Engineering Laboratory for Pollution Control and Waste Utilization in Livestock and Poultry Production, Institute of Subtropical Agriculture, Chinese Academy of Sciences, Changsha, Hunan, China; b College of Advanced Agricultural Sciences, University of Chinese Academy of Sciences, Beijing, China; c College of Animal Science and Technology, Hunan Agricultural University, Changsha, Hunan, China; d School of Biology and Food Engineering, Fuyang Normal University, Fuyang, Anhui, China

**Keywords:** Chinese herb, egg quality, nutritional values, ultrafine powder, Xinyang black-feather hens

## Abstract

This study evaluates the effects of dietary Chinese herb ultrafine powder (CHUP) supplementation in late-phase laying hens on the quality and nutritional values of eggs. A total of 576 Xinyang black-feather laying hens (300-day-old) were randomly allocated into eight groups for a 120-day feeding trial. Each group contained eight replicates with nine hens per replicate. The experimental groups included the control (basal diet) and different levels of CHUP groups (details in ‘Materials and methods’). The results showed that the eggshell strength was increased (*p* < 0.05) in the L, LF, L-LF, L-T, and LF-T groups on day 60 of the trial. In addition, the plasma estradiol level in the L-LF, LF-T, and L-LF-T groups and unsaturated fatty acids concentrations in egg yolk of the CHUP groups (except LF-T group) were increased, whereas total cholesterol (T, L-LF, L-T, and L-LF-T groups) in egg yolk and the atherogenicity (T, L-T, and L-LF-T groups) and thrombogenicity (T, L-LF, L-T, and L-LF-T groups) indexes were decreased (*p* < 0.05) on day 60 of the trial compared with the control group. Moreover, bitter amino acids in egg albumen were decreased (*p* < 0.05) in the L-LF group on day 60 and the L-LF-T group on day 120 of the trial. Collectively, these findings indicate that dietary CHUP supplementation could improve eggshell quality and increase plasma reproductive hormone, fatty acid and amino acid composition, and nutritional values of eggs, especially L-LF and L-LF-T.

## Introduction

Eggs are a highly nutritious food that contains the majority of the essential nutrients for human health. In comparison to other protein sources from foods, eggs are high in vitamin A, folate, biotin, iodine, and cholesterol while low in energy. Research evidence reveals that the consumption of eggs on a regular and long-term basis contributes to a more nutritious diet (Ruxton et al. 2010). However, egg production performance, quality, and nutritional composition are influenced by the age and production level of laying hens (Skřivan et al. 2010). The quality of eggshells decrease with the progressed age of laying hens, which causes the rate of broken eggs during the late laying stage (Bennett 1992). Therefore, improving laying performance and egg quality is critical during the late laying stage. Hence, it is necessary to investigate whether feed additives during this stage can enhance laying performance, egg quality, and nutritional values of eggs.

Studies on herbal feed additives have gained interest in recent years due to antibiotic-free alternative strategies. As nutritional supplements, Chinese herbs have been shown to boost laying performance, egg quality, plasma hormone levels (Zhang et al. 2021), eggshell strength, and anti-oxidative enzymes (Xiao et al. 2019) in poultry. Most traditional Chinese herbs are currently used as feed additives by drying and crushing raw materials (Zhu et al. 2022) or directly adding Chinese herb extracts (Chen et al. 2022). However, these traditional methods have certain limitations, such as the bioactive ingredients, which are not completely absorbed by the body due to the particle size of the formulated herb powder. In addition, the large particle size of the formulated herb powder failed to achieve therapeutic efficacy or meet the compound formula’s preparation conditions. Using modern ultrafine powder technology, the application of Chinese herb ultrafine powder (CHUP) provides a new effective strategy for Chinese herbs (Li et al. 2019). The CHUP showed some advantages, such as the solubility of the bioactive ingredients increases due to the increase in surface area, which is conducive to the gastrointestinal tract’s absorption and the bioavailability of the herb. Ultrafine herb power has been shown to be more effective than traditionally processed herbs when compared with their efficacy (Wang et al. 2011). However, little information exists regarding CHUP and its effects on nutritional values and egg quality in laying hens.

In accordance with the ‘sovereign, minister, assistant, and courier’ theory, *Leonuri herba* (L), *Ligustri lucidi fructus* (LF), and *Taraxaci herba* (T) were prepared into CHUP. The L is known as the holy herb of gynecology and has bitter and pungent tastes while activating blood, resolving stasis, promoting urination, and dispersing swelling (Qiao et al. 2019). The LF was found more effective for nourishing liver and kidney, tonifying Yin, and benefiting essence (Cai et al. 2011), which combined with L for strengthening healthy Qi and resolving stasis. The T is a heat-clearing herb for detoxifying functions, swell-reducing, and mass-resolving effects (Yang et al. 2016), which could enhance the medicinal properties of L. Therefore, the LF and T were employed as excellent herbs together. Furthermore, previous studies found that diets supplemented with 0.25% LF improved the laying performance and egg quality of Hy-Line brown hens compared with diets supplemented with 0.50% LF (Li et al. 2017). Therefore, we speculated that diets supplemented with CHUP might improve the quality and nutritional values of eggs of laying hens. Thus, this study evaluated the effects of dietary CHUP on the quality and nutritional values of eggs of laying hens during the late laying stage, and the outcomes would provide an effective and low-cost dietary formulation method for improving laying hen production.

## Materials and methods

### Experimental birds, diets, and management

A total of 576 healthy 300-day-old laying hens (Xinyang black-feather) with similar laying performance were randomly allocated to eight groups (eight replicate hens per group and nine hens per replicate with three hens per cage). The birds in the control group were provided a basal diet, and the birds in the treatment groups were provided a basal diet supplemented with 0.50% *Leonuri herba* (L group), 0.25% *Ligustri lucidi fructus* (LF group), 0.25% *Taraxaci herba* (T group), 0.50% L + 0.25% LF (L-LF group), 0.50% L + 0.25% T (L-T group), 0.25% LF + 0.25% T (LF-T group), or 0.50% L + 0.25% LF + 0.25% T (L-LF-T group), respectively. The feeding trial lasted 120 days. Dietary additives (CHUP) were obtained from Kangxing Pharmaceutical Co. Ltd. (Zhengzhou, China). The hens were reared in four-layer ladder cages. The experimental hens had access to *ad libitum* feed and water. The room temperature was maintained at 25 °C with a 16 h/8 h light/dark cycle. The hens were fed twice daily at 07:30 and 16:00 h with their respective diets throughout the experimental period, and water was available by a nipple-type drinker. All experimental diets were formulated to meet or exceed the layer-feeding standard (NY/T 33-2004). The composition and nutrient levels of the basal diet are presented in Table 1.

**Table 1.** Ingredients and nutrient composition of the basal diet, % air-dried.

Ingredients	Content	Nutrients^b^	Content
Corn	62.59	Metabolic energy (MJ/kg)	11.25
Soybean meal	23.88	Crude protein	14.23
Limestone powder	7.94	Ether extract	9.66
Soybean oil	0.49	Calcium	3.51
Methionine	0.10	Phosphorus	0.34
Premix^a^	5.00	Lysine	0.71
Total	100.00	Methionine	0.37

^a^ The premix provided the following per kg of diets: vitamin A, 140,000 IU; vitamin D_3_, 50,000 IU; vitamin E, 480 mg; vitamin Κ, 3.18 g; vitamin B_1_, 63 mg; vitamin B_2_, 200 mg; vitamin B_6_, 140 mg; vitamin B_12_, 0.7 mg; nicotinic acid, 1000 mg; D-pantothenic acid, 500 mg; folic acid, 50 mg; D-biotin, 5.0 mg; choline chloride, 900 mg; Fe, 2.0 g; Cu, 0.3 g; Mn, 1.8 g; Zn, 2.0 g; I, 70 mg; Se, 9 mg; and phytase, 3000 IU.

^b^ Crude protein and ether extract were measured values, and the others were calculated values.

### Sample collection

Three eggs from each replicate were randomly collected on days 30, 60, 90, and 120 of the trial to determine the egg quality. Blood samples (5 mL) were collected randomly in tubes anticoagulated with sodium heparin *via* the wing vein from one bird per replicate on days 60 and 120 of the trial. Then, blood samples were centrifuged at 3500 × *g* for 10 min at 4 °C to obtain the plasma and stored at −20 °C for reproductive hormone and total cholesterol assays. Egg yolk and albumen were sampled into sterile centrifuge tubes and stored at −80 °C until further metabolite analyses, including medium- and long-chain fatty acid and amino acid.

### Laying performance

The egg production and egg weight were recorded daily. The feed intake of each replicate was measured weekly in accordance with the farm performance and measurement for poultry (NY/T 823-2004). Average egg production, average egg weight, average daily feed intake (ADFI), and feed conversion ratio (FCR) were calculated during 1–30, 31–60, 61–90, 91–120, and 1–120 days of the trial.

### Egg quality parameters

Egg albumen height, Haugh unit, and yolk color were detected using a Multifunctional Egg Quality detector (EA-01, Tenovo International Co. Ltd., Beijing, China). Eggshell strength was measured by Eggshell Strength Tester (KQ-1A, Tenovo International Co. Ltd., Beijing, China), and the eggshell thickness was the average of the thickness of the sharp end, blunt end, and middle section of the eggshell (the inner shell membrane was removed from eggshell) using an Eggshell Thickness Tester (ESTG-01, Tenovo International Co. Ltd., Beijing, China). The transverse to longitudinal diameter ratio was measured using a Vernier caliper to calculate the eggshape index.

### Plasma reproductive hormones and the total cholesterol in plasma and egg yolk

The plasma levels of the reproductive hormones, including follicle-stimulating hormone (FSH), luteinizing hormone (LH), and estradiol (E2) and the total cholesterol were determined using enzyme-linked immunosorbent assays (ELISA) kits (Jiangsu Industrial Co. Ltd., Yancheng, China) according to the manufacturer’s protocols. The total cholesterol level in egg yolk was determined using the total cholesterol kit (Nanjing Jianjian Biotechnology Co. Ltd., Nanjing, China) following the manufacturer’s instructions.

### Fatty acid composition and nutritional values of egg yolk

Egg yolk (100 mg) samples were transferred into 2 mL EP tubes and homogenized by the Vortex Oscillator (Hunan Xiangyi Laboratory Instrument Development Co. Ltd., Changsha, China). The samples were vortexed with 800 µL of 50% acetonitrile water for 1 min, and the supernatants (400 µL) were collected after centrifuging at 12,000 × *g* and 4 °C for 15 min. The supernatants were vortexed for 1 min with 200 µL 3-nitrophenylhydrazine (200 mM) and 200 µL EDC (120 mM; containing 6% pyridine and 400 ng/mL of acetic acid-d4), respectively. The mixture solutions were centrifuged at 12,000 × *g* and 4 °C for 15 min after being reacted at 40 °C for 1 h with shaking each 5-min to obtain supernatants. Finally, the supernatants were filtered through 0.22-μm membrane and diluted 10 times with 50% acetonitrile water (containing 100 ng/mL of internal standard) to obtain the prepared samples for liquid chromatography-tandem mass spectrometry (LC-MS) analysis.

Saturated fatty acid (SFA), monounsaturated fatty acid (MUFA), polyunsaturated fatty acid (PUFA), n-3 PUFA, n-6 PUFA, n-6/n-3 PUFA, and unsaturated fatty acid (UFA) were calculated based on the common fatty acid content of the egg yolk. The following formulas were used to calculate the nutritional values of the egg yolk, including atherogencity index (AI), thrombogenicity index (TI), desirable hypocholes-terolemic fatty acids (DHFA), ratio of hypercholesterolemic to hypercholesterolemic fatty acids (HH), and hypercholesterolemic saturated fatty acids (HSFA) (Zhang et al. 2022):

$$\text{AI=}4\times \frac{\text{C14:0+C16:0}}{\sum \text{MUFA+}\sum \left(n-6\right)+\sum \text{(n}-\text{3)}}$$

$$\text{TI=}\frac{\text{C14:0+C16:0+C18:0}}{\begin{array}{l} 0\text{.5}0\times \sum \text{MUFA+0.5}0\times \sum \left(n-6\right)+3\times \sum \left(n-3\right)\\ +\sum \left(n-3\right)\text{/}\sum \left(n-6\right) \end{array}}$$

DHFA = MUFA + PUFA + C18:0

$$\text{HH=}\frac{\text{C18:1+C18:2n}-\text{6+C18:3n}-3}{\text{C14:0+C16:0}}$$

HSFA = C14:0+ C16:0

### Amino acid composition and nutritional values of egg albumen

Egg albumen samples were aliquoted into 10 mL EP tubes after thawing at 4 °C, and then transferred into 10 mL ampoule bottles after homogenizing by the Vortex Oscillator (Hunan Xiangyi Laboratory Instrument Development Co. Ltd., Changsha, China). The homogenised samples (200 µL) were fully shaken with 8 mL of 6 M hydrochloric acid for 1 min, sonicated at 4 °C for 30 min, and then allowed to stand at −60 °C for 10 min. The samples were connected to a vacuum pump, sealed, and then transferred into a drying box at 110 °C for 22 h. The hydrolyzed samples (1 mL) were put into a lyophilizer and freeze-dried for 60 min. The freeze-dried samples were dissolved with 50% acetonitrile water (0.2 mL; containing 100 ng/mL of L-tryptophan-d5 internal standard). The mixture solutions were sonicated at 4 °C for 10 min and centrifuged at 12,000 × *g* for 10 min after shaking for 1 min. Finally, the supernatants were filtered through 0.22-μm membrane to obtain the prepared samples for LC-MS analysis. The Waters Acquity UPLC (Waters, Milford, MA, USA) and AB SCIEX QTRAP® 5500 (AB SCIEX, Framingham, MA, USA) were used to conduct the LC-MS analysis.

The following formulas were used to calculate the nutritional values of the amino acid, including acidic amino acid (AAA), aromatic amino acid (ARAA), basic amino acid (BAA), branched-chain amino acid (BCAA), essential amino acid (EAA), and sulphur-containing amino acid (SAA) (Baki et al. 2021):

AAA =aspartic acid +glutamic acid

ARAA =phenylalanine +tyrosine

BAA =histidine +lysine +arginine

BCAA =leucine +isoleucine +valine

$$\begin{array}{c} \text{EAA}=\text{histidine}+\text{lysine}+\text{phenylalanine}+\text{methionine}\\ +\text{threonine}+\text{leucine}+\text{isoleucine}+\text{valine}\\ +\text{arginine}+\text{tryptophan} \end{array}$$

SAA =methionine +cystine

### Statistical analysis

The experimental unit for all egg-related data in this test was the cage, and the other data was the individual hen. Statistical data analyses were conducted by one-way analysis of variance (ANOVA) using SPSS (version 26.0) software. All data were checked for normal distribution, and then Duncan’s multiple range test was subjected to evaluate statistical significance among different treatment groups. Data are presented as means with their standard error of the mean (SEM). Statistical significant differences among different groups were considered when *p*** **<** **0.05, and trends were considered when 0.05 *≤* *p*** ***<*** **0.10. The SIMCA 14.1 was used to perform principal components analysis (PCA) and orthogonal partial least squares-discriminant analysis (OPLS-DA). Spearman’s correlation analysis was performed to analyze correlations between yolk color, plasma reproductive hormones, total cholesterol in egg yolk, and fatty acid and amino acid content in egg using the Origin 2021 software package.

## Results

### Effects of CHUP on laying performance

The effects of dietary CHUP supplementation on laying performance are shown in Table 2. During 1 − 30 days of the trial, the ADFI in the L-LF-T group was lower (*p*** **<** **0.05) than that in the control group, and it was lower (*p*** **<** **0.05) in the T, L-LF, L-T, and L-LF-T groups compared with the LF-T group, as well as in the T and L-LF-T groups compared with the L and LF groups. Additionally, ADFI displayed a decreasing trend (*p*** **=** **0.096) in the T, L-LF, L-T, and L-LF-T groups compared with the control group during 31 − 60 days of the trial. Moreover, ADFI was higher (*p*** **<** **0.05) in the L and LF-T groups compared with the L-T group during 1 − 30 and 1 − 120 days of the trial. However, there were no significant changes in egg production, egg weight, and FCR throughout the trial (*p*** **>** **0.05).

**Table 2.** Effects of Chinese herb ultrafine power (CHUP) on laying performance of laying hens.

Items	Dietary groups								SEM	*P*-values
	Control	L	LF	T	L-LF	L-T	LF-T	L-LF-T		
Days 1–30 of the trial										
ADFI (g/d)	113.05^abcd^	115.17^ab^	114.39^abc^	108.87^de^	111.47^bcde^	109.46^cde^	117.42^a^	107.72^e^	0.674	0.001
Egg production (%)	84.72	88.38	85.51	84.17	83.10	84.58	87.73	85.66	0.007	0.518
Egg weight (g)	51.42	51.87	50.85	51.94	51.46	51.70	51.41	51.58	0.121	0.486
FCR (g/g)	2.60	2.51	2.61	2.49	2.64	2.52	2.47	2.61	0.020	0.230
Days 31–60 of the trial										
ADFI (g/d)	109.06	111.35	110.51	107.78	107.77	105.81	112.66	106.81	0.649	0.096
Egg production (%)	84.91	85.70	81.20	83.79	81.99	82.83	85.14	83.45	0.006	0.596
Egg weight (g)	51.83	52.57	51.32	52.16	51.98	51.50	51.94	51.73	0.143	0.491
FCR (g/g)	2.48	2.48	2.64	2.a47	2.56	2.49	2.45	2.55	0.020	0.253
Days 61–90 of the trial										
ADFI (g/d)	100.47	104.42	105.65	101.97	103.51	97.52	104.14	101.85	0.688	0.077
Egg production (%)	79.41	82.79	80.45	80.86	79.48	79.03	80.99	78.76	0.008	0.919
Egg weight (g)	52.33	52.77	51.39	52.45	51.89	51.64	52.00	52.19	0.159	0.445
FCR (g/g)	2.42	2.40	2.51	2.42	2.52	2.39	2.49	2.43	0.025	0.808
Days 91–120 of the trial										
ADFI (g/d)	101.80	100.93	100.24	100.83	99.95	91.70	100.57	99.01	0.953	0.242
Egg production (%)	76.80	82.13	78.49	78.69	79.90	73.85	79.57	76.79	0.007	0.135
Egg weight (g)	52.28	52.96	51.40	52.28	52.28	51.65	52.39	52.87	0.166	0.258
FCR (g/g)	2.54	2.32	2.42	2.32	2.46	2.46	2.48	2.58	0.029	0.247
Days 1–120 of the trial										
ADFI (g/d)	106.50^ab^	108.51^a^	108.81^a^	104.63^ab^	105.69^ab^	102.11^b^	109.32^a^	104.39^ab^	0.642	0.049
Egg production (%)	81.79	84.95	82.41	82.11	80.19	80.39	84.56	81.12	0.006	0.512
Egg weight (g)	51.93	52.50	51.63	52.19	51.87	51.54	51.89	51.79	0.150	0.835
FCR (g/g)	2.51	2.43	2.52	2.44	2.58	2.47	2.47	2.53	0.021	0.672

Data are expressed as means with their SEM (*n* = 8). ^a-e^ Different letters indicate differences (*p* < 0.05) between mean values tested by Duncan’s multiple range test. ADFI, Average daily feed intake; FCR, Feed conversion ratio. Control group, fed a basal diet; L group, basal diet supplemented with 0.50% *Leonuri herba*; LF group, basal diet supplemented with 0.25% *Ligustri lucidi fructus*; T group, basal diet supplemented with 0.25% *Taraxaci herba*; L-LF group, basal diet supplemented with 0.50% L + 0.25% LF; L-T group, basal diet supplemented with 0.50% L + 0.25% T; LF-T group, basal diet supplemented with 0.25% LF + 0.25% T; L-LF-T group, basal diet supplemented with 0.50% L + 0.25% LF + 0.25% T.

### Effects of CHUP on egg quality

The effects of dietary CHUP on laying hens’ egg quality are presented in Table 3. On day 60 of the trial, CHUP supplementation (all treatment groups) increased (*p*** **<** **0.05) the eggshell thickness compared with the control group. The eggshell strength was higher (*p*** **<** **0.05) in the L, LF, L-LF, L-T, and LF-T groups, whereas the eggshape index was lower (*p*** **<** **0.05) in the L, L-LF, and LF-T groups, as well as the eggshell ratio in the L group on day 60 of the trial, when compared with the control group. However, eggshell ratio was higher (*p*** **<** **0.05) in the LF, T, L-LF, and LF-T groups compared with the L and L-LF-T groups, as well as in the L-T group compared with the L group on day 60 of the trial. In addition, yolk color was higher (*p*** **<** **0.05) in the L, LF, and L-LF-T groups compared with the L-T group, as well as in the L group compared with the T group, whereas the eggshape index was lower (*p*** **<** **0.05) in the L, L-LF, and LF-T groups compared with the L-LF-T group and the eggshell thickness was higher (*p*** **<** **0.05) in the L group compared with the L-LF group on day 60 of the trial. On day 90 of the trial, the eggshape index was higher (*p*** **<** **0.05) in the L-T and L-LF-T groups, while yolk color was higher (*p*** **<** **0.05) in the L and LF-T groups compared with the control group. However, the eggshape index was higher (*p*** **<** **0.05) in the L, T, L-T, and L-LF-T groups than the LF-T group, as well as in the L-T group than the LF group, and yolk color was higher (*p*** **<** **0.05) in the L, LF, L-T, and LF-T groups than the T group. On day 120 of the trial, CHUP supplementation increased (*p*** **<** **0.05) the eggshell strength (except the L and L-T groups) compared with the control group. However, the eggshell thickness in the L-LF, L-T, LF-T, and L-LF-T groups, eggshape index in the LF-T group, and yolk color in the L, T, L-LF, L-T, and L-LF-T groups were lower (*p*** **<** **0.05) compared with the control group. Moreover, albumen height was lower (*p*** **<** **0.05) in the L, LF, T, L-LF, and L-LF-T groups compared with the LF-T group, eggshell thickness was higher (*p*** **<** **0.05) in the L-LF-T group compared with the L-LF, L-T, and LF-T groups, and yolk color was higher (*p*** **<** **0.05) in the T and L-LF-T groups compared with the L and L-LF groups. Haugh unit displayed an increasing trend in the L, T, L-T, and L-LF-T groups (*p*** **=** **0.089) on day 30 of the trial, as well as in the LF-T group (*p*** **=** **0.095) on day 120 of the trial, when compared with the control group. However, albumen ratio and yolk ratio did not significantly change throughout the trial (*p*** **>** **0.05).

**Table 3.** Effects of Chinese herb ultrafine power (CHUP) on egg quality of laying hens.

Items	Dietary groups								SEM	*P*-values
	Control	L	LF	T	L-LF	L-T	LF-T	L-LF-T		
Day 30 of the trial										
Albumen height (mm)	4.09	4.80	3.95	4.28	4.19	4.90	4.05	4.36	0.098	0.141
Albumen ratio (%)	57.59	57.59	56.68	56.63	56.65	56.79	56.85	57.27	0.002	0.742
Eggshape index	1.32	1.33	1.31	1.32	1.31	1.33	1.32	1.32	0.003	0.674
Eggshell ratio (%)	10.99	10.91	10.66	11.07	10.73	11.10	10.98	10.91	0.001	0.514
Eggshell strength (N)	39.43	42.38	41.12	42.68	40.79	41.99	39.42	40.37	0.671	0.891
Eggshell thickness (mm)	0.37	0.37	0.36	0.37	0.37	0.38	0.38	0.38	0.002	0.522
Haugh unit	61.94	71.16	57.27	63.68	61.94	68.91	60.88	64.64	1.182	0.089
Yolk color	11.90	11.77	11.71	11.67	11.62	11.71	11.63	11.83	0.043	0.741
Yolk ratio (%)	31.65	31.97	32.66	32.52	32.99	32.49	32.16	31.72	0.002	0.388
Day 60 of the trial										
Albumen height (mm)	3.84	4.28	3.69	4.03	3.93	4.24	4.48	4.75	0.113	0.289
Albumen ratio (%)	56.18	57.41	55.89	56.25	56.19	56.51	55.95	56.87	0.002	0.271
Eggshape index	1.34^a^	1.30^b^	1.33^ab^	1.33^ab^	1.30^b^	1.33^ab^	1.31^b^	1.34^a^	0.004	0.012
Eggshell ratio (%)	10.66^ab^	10.04^c^	10.87^a^	10.97^a^	10.96^a^	10.67^ab^	10.01^a^	10.37^bc^	0.001	<0.001
Eggshell strength (N)	30.90^b^	37.50^a^	40.48^a^	36.42^ab^	36.93^a^	41.15^a^	38.97^a^	36.26^ab^	0.743	0.017
Eggshell thickness (mm)	0.33^c^	0.37^a^	0.36^ab^	0.36^ab^	0.36^b^	0.37^ab^	0.37^ab^	0.36^ab^	0.002	<0.001
Haugh unit	59.98	62.44	57.99	61.16	59.31	63.13	63.01	68.08	1.223	0.593
Yolk color	12.25^abc^	12.83^a^	12.71^ab^	12.13^bc^	12.42^abc^	11.98^c^	12.50^abc^	12.67^ab^	0.074	0.037
Yolk ratio (%)	33.17	32.56	33.27	32.58	32.85	33.02	33.04	33.77	0.001	0.910
Day 90 of the trial										
Albumen height (mm)	3.94	4.42	3.77	3.79	4.07	4.43	4.58	4.32	0.110	0.432
Albumen ratio (%)	57.69	55.75	56.02	56.52	55.58	55.67	56.35	56.35	0.002	0.227
Eggshape index	1.31^cd^	1.33^abc^	1.32^bcd^	1.33^abc^	1.33^abcd^	1.35^a^	1.30^d^	1.35^ab^	0.004	0.005
Eggshell ratio (%)	10.69	11.01	11.06	10.57	10.98	10.91	10.88	11.06	0.001	0.410
Eggshell strength (*N*)	36.77	36.69	32.47	38.00	36.29	36.18	34.16	34.97	0.586	0.388
Eggshell thickness (mm)	0.37	0.37	0.36	0.35	0.36	0.36	0.36	0.36	0.002	0.374
Haugh unit	60.36	63.80	56.88	58.20	63.25	65.79	65.07	62.60	1.288	0.625
Yolk color	12.05^bc^	12.67^a^	12.46^ab^	11.90^c^	12.23^abc^	12.46^ab^	12.71^a^	12.21^abc^	0.065	0.010
Yolk ratio (%)	31.72	33.24	32.97	32.83	33.81	32.36	32.78	32.59	0.002	0.278
Day 120 of the trial										
Albumen height (mm)	3.93^ab^	3.47^b^	3.55^b^	3.34^b^	3.43^b^	4.08^ab^	4.64^a^	3.68^b^	0.097	0.011
Albumen ratio (%)	56.30	56.33	56.49	56.65	56.71	57.00	56.86	56.91	0.002	0.944
Eggshape index	1.35^ab^	1.35^ab^	1.35^b^	1.34^b^	1.32^b^	1.34^b^	1.30^c^	1.38^a^	0.004	<0.001
Eggshell ratio (%)	10.44	10.51	10.49	10.72	10.33	10.08	10.36	10.76	0.001	0.076
Eggshell strength (*N*)	32.69^b^	32.27^b^	37.47^a^	37.79^a^	38.10^a^	34.51^ab^	38.65^a^	37.85^a^	0.609	0.020
Eggshell thickness (mm)	0.37^a^	0.36^ab^	0.36^ab^	0.36^ab^	0.33^c^	0.33^c^	0.33^c^	0.35^b^	0.003	<0.001
Haugh unit	62.07	59.58	57.95	54.96	55.70	62.64	66.78	58.50	1.050	0.095
Yolk color	12.21^a^	11.28^d^	12.08^a^	11.60^bc^	11.29^d^	11.44^cd^	12.10^a^	11.76^b^	0.057	<0.001
Yolk ratio (%)	33.27	33.17	32.98	32.92	33.14	33.17	33.88	32.34	0.001	0.757

Data are expressed as means with their SEM (*n* = 8). ^a-d^ Different letters indicate differences (*p* < 0.05) between mean values tested by Duncan’s multiple range test. Control group, fed a basal diet; L group, basal diet supplemented with 0.50% *Leonuri herba*; LF group, basal diet supplemented with 0.25% *Ligustri lucidi fructus*; T group, basal diet supplemented with 0.25% *Taraxaci herba*; L-LF group, basal diet supplemented with 0.50% L + 0.25% LF; L-T group, basal diet supplemented with 0.50% L + 0.25% T; LF-T group, basal diet supplemented with 0.25% LF + 0.25% T; L-LF-T group, basal diet supplemented with 0.50% L + 0.25% LF + 0.25% T.

### Effects of CHUP on plasma reproductive hormones and the total cholesterol in plasma and egg yolk

The levels of FSH, LH, E2, and the total cholesterol in plasma (TCP) and egg yolk (TCEY) were measured on days 60 and 120 of the trial to evaluate the effects of dietary CHUP in laying hens (Table 4**)**. On day 60 of the trial, the plasma E2 level in the L-LF, LF-T, and L-LF-T groups was higher (*p*** **<** **0.05) than that in the control group. The plasma LH level was higher (*p*** **<** **0.05) in the LF and L-T groups, whereas it was lower (*p*** **<** **0.05) in the LF-T group, when compared with the control group. The plasma FSH level was higher (*p*** **<** **0.05) in the LF, T, L-LF, and L-T groups, while the total cholesterol level was lower (*p*** **<** **0.05) in the T, L-LF, L-T, and L-LF-T groups, when compared with the control group. In addition, the plasma FSH level was higher (*p*** **<** **0.05) in the L-T group compared with the LF, T, and L-LF groups. On day 120 of the trial, the plasma FSH level was higher (*p*** **<** **0.05) in the L-T and L-LF-T groups than that in the control group, while it was higher (*p*** **<** **0.05) in the L-LF-T group compared with the L and T groups. However, there were no significant differences in E2, LH, and the total cholesterol on day 120 of the trial (*p* > 0.05).

**Table 4.** Effects of Chinese herb ultrafine power (CHUP) on plasma reproductive hormones and total cholesterol in plasma and egg yolk of laying hens.

Items	Dietary groups								SEM	*P*-values
	Control	L	LF	T	L-LF	L-T	LF-T	L-LF-T		
Day 60 of the trial										
E2 (pg/mL)	401.40^cd^	385.74^d^	418.90^cd^	445.66^bc^	497.30^a^	407.99^cd^	488.64^ab^	486.47^ab^	7.671	<0.001
FSH (mIU/mL)	18.34^cd^	17.39^cd^	20.53^b^	20.67^b^	20.58^b^	22.28^a^	18.62^c^	17.06^d^	0.272	<0.001
LH (mIU/mL)	17.04^bc^	16.65^bc^	18.60^a^	17.58^ab^	17.46^abc^	18.64^a^	15.33^d^	16.14^cd^	0.197	<0.001
TCEY (mg/g)	8.56^a^	7.90^abc^	7.76^abcd^	6.75^d^	6.82^d^	7.32^bcd^	8.14^ab^	6.98^cd^	0.136	0.002
TCP (mmol/L)	3.08	2.63	3.76	3.83	3.87	3.71	4.03	4.35	0.159	0.180
Day 120 of the trial										
E2 (pg/mL)	405.59	398.67	399.64	419.55	424.70	408.87	411.96	413.83	4.183	0.775
FSH (mIU/mL)	16.79^c^	17.21^bc^	17.52^abc^	16.36^c^	18.16^abc^	18.87^ab^	18.07^abc^	19.36^a^	0.239	0.014
LH (mIU/mL)	16.75	16.91	16.95	16.79	17.22	18.17	17.47	18.00	0.148	0.108
TCEY (mg/g)	9.93	10.46	10.10	9.64	9.93	10.35	10.43	10.12	0.122	0.711
TCP (mmol/L)	2.21	3.94	3.79	2.68	2.19	3.03	3.34	2.88	0.167	0.085

Data are expressed as means with their SEM (*n* = 8). ^a-d^ Different letters indicate differences (*p* < 0.05) between mean values tested by Duncan’s multiple range test. FSH, follicle-stimulating hormone; LH, luteinizing hormone; E2, estradiol; TCP, total cholesterol in plasma; TCEY, total cholesterol in egg yolk. Control group, fed a basal diet; L group, basal diet supplemented with 0.50% *Leonuri herba*; LF group, basal diet supplemented with 0.25% *Ligustri lucidi fructus*; T group, basal diet supplemented with 0.25% *Taraxaci herba*; L-LF group, basal diet supplemented with 0.50% L + 0.25% LF; L-T group, basal diet supplemented with 0.50% L + 0.25% T; LF-T group, basal diet supplemented with 0.25% LF + 0.25% T; L-LF-T group, basal diet supplemented with 0.50% L + 0.25% LF + 0.25% T.

### Effects of CHUP on fatty acid composition in egg yolk

**Figure 1.**
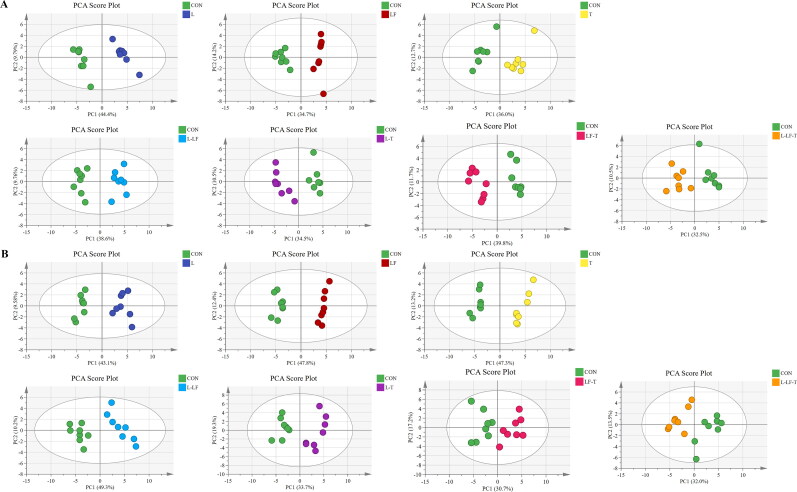
Principal component analysis (PCA) scores plots. Fatty acid targeted metabolomics of the egg yolk on days 60 (A) and 120 (B) of the trial. Control group (CON), fed a basal diet; L group, basal diet supplemented with 0.50% *Leonuri herba*; LF group, basal diet supplemented with 0.25% *Ligustri lucidi fructus*; T group, basal diet supplemented with 0.25% *Taraxaci herba*; L-LF group, basal diet supplemented with 0.50% L + 0.25% LF; L-T group, basal diet supplemented with 0.50% L + 0.25% T; LF-T group, basal diet supplemented with 0.25% LF + 0.25% T; L-LF-T group, basal diet supplemented with 0.50% L + 0.25% LF + 0.25% T.

**Figure 2.**
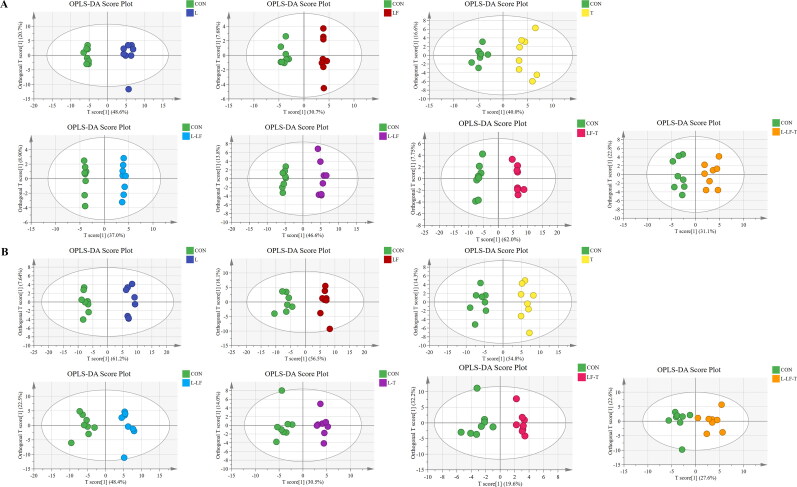
Orthogonal partial least squares-discriminant analysis (OPLS-DA) scores plots of fatty acid in egg yolk on days 60 (A) and 120 (B) of the trial. Control group (CON), fed a basal diet; L group, basal diet supplemented with 0.50% *Leonuri herba*; LF group, basal diet supplemented with 0.25% *Ligustri lucidi fructus*; T group, basal diet supplemented with 0.25% *Taraxaci herba*; L-LF group, basal diet supplemented with 0.50% L + 0.25% LF; L-T group, basal diet supplemented with 0.50% L + 0.25% T; LF-T group, basal diet supplemented with 0.25% LF + 0.25% T; L-LF-T group, basal diet supplemented with 0.50% L + 0.25% LF + 0.25% T.

The PCA analysis results of fatty acid are shown in Figure 1. On days 60 and 120 of the trial, the profile of fatty acid composition in different treatment groups displayed distinct separations between the treatment and control groups, indicating that different dietary CHUP supplementation influenced the fatty acid composition in egg yolks. The OPLS-DA analysis was conducted to emphasize the differences between the groups for more clarification (Figure 2). The OPLS-DA results supported the same trend as the PCA results regarding fatty acid composition.

The composition of fatty acid in egg yolk on day 60 of the trial is presented in Table 5. The content of C18:0, C18:1n-9t, C18:2n-6, C18:3n-3, C18:3n-6, C20:1, and C20:1t were higher (*p*** **<** **0.05) in the egg yolk of all treatment groups compared with the control group; notably, all of these fatty acids were higher (*p*** **<** **0.05) in the L-LF-T group compared with the other treatment groups. The L, LF, T, L-LF, and L-T groups had higher (*p*** **<** **0.05) SFAs compared with the control group. In addition, the C14:0 and C18:0 content was higher (*p*** **<** **0.05) in the T, L-LF, L-T, LF-T, and L-LF-T groups (particularly in the L-LF-T group), whereas the C15:0 content was lower (*p*** **<** **0.05) in the L, LF, T, L-LF, and LF-T groups, when compared with the control group. All treatment groups had higher MUFAs, the L, L-LF, L-T, and L-LF-T groups had higher n-6/n-3 PUFA ratio, PUFAs, and UFAs, whereas the L-LF-T group was the major contributor. Compared with the control group, the AI was decreased (*p*** **<** **0.05) in the T, L-T, and L-LF-T groups, and the TI was decreased (*p*** **<** **0.05) in the T, L-LF, L-T, and L-LF-T groups. The HSFA in the L group, as well as DHFA in all treatment (except the LF-T group) groups, were higher (*p*** **<** **0.05) compared with the control group.

**Table 5.** Effects of Chinese herb ultrafine power (CHUP) on fatty acid composition in egg yolk (ng/mg of egg yolk, wet basis) of laying hens on day 60 of the trial.

Items	Dietary groups								SEM	*P*-values
	Control	L	LF	T	L-LF	L-T	LF-T	L-LF-T		
C6:0	1.33^cd^	3.85^a^	2.83^b^	1.66^c^	1.47^c^	1.04^cd^	0.67^d^	0.94^cd^	0.152	<0.001
C7:0	0.21^ab^	0.37^ab^	0.23^ab^	0.43^a^	0.33^ab^	0.22^ab^	0.13^b^	0.43^a^	0.028	0.045
C11:0	0.38	0.11	0.27	0.22	0.47	0.35	0.37	0.38	0.030	0.075
C12:0	0.22^b^	8.26^a^	6.24^a^	8.49^a^	8.73^a^	4.79^ab^	0.23^b^	0.23^b^	0.702	<0.001
C13:0	1.44	1.71	1.32	1.36	1.71	1.64	0.81	1.17	0.122	0.604
C14:0	1.41^e^	1.45^e^	1.28^f^	1.67^d^	2.70^b^	1.95^c^	1.65^d^	3.12^a^	0.080	<0.001
C15:0	0.53^a^	0.25^f^	0.28^ef^	0.34^de^	0.44^bc^	0.50^ab^	0.40^cd^	0.48^ab^	0.014	<0.001
C16:0	31.37^bc^	45.54^a^	34.27^b^	27.86^bcd^	36.19^b^	29.86^bcd^	24.53^cd^	21.27^d^	1.303	<0.001
C17:0	0.13^ab^	0.14^a^	0.12^b^	0.13^ab^	0.14^ab^	0.13^b^	0.12^b^	0.13^ab^	0.002	0.038
C18:0	17.64^e^	19.92^d^	23.87^c^	23.50^c^	27.54^b^	28.50^b^	23.47^c^	34.36^a^	0.669	<0.001
C20:0	0.90^ab^	0.37^c^	1.07^ab^	0.87^b^	0.62^bc^	1.32^a^	1.03^ab^	0.87^b^	0.059	0.001
C21:0	12.94	16.81	13.74	14.24	14.99	12.57	10.73	15.02	0.530	0.151
C22:0	0.99^b^	2.59^ab^	1.91^ab^	4.15^a^	1.14^b^	0.80^b^	4.02^a^	1.00^b^	0.343	0.034
C23:0	8.85^abc^	9.79^ab^	9.73^ab^	10.81^a^	8.18^bc^	7.93^bc^	7.17^c^	9.71^ab^	0.272	0.010
C24:0	14.61^bcd^	8.87^e^	13.84^cde^	20.11^a^	19.27^ab^	16.52^abc^	10.91^de^	13.66^cde^	0.746	<0.001
ΣSFA	89.07^cd^	117.98^ab^	114.04^ab^	109.20^ab^	123.58^a^	107.93^ab^	83.20^d^	102.55^bc^	2.471	<0.001
C15:1	0.22^de^	0.61^a^	0.50^b^	0.32^cd^	0.35^c^	0.27^cde^	0.16^e^	0.21^e^	0.022	<0.001
C15:1t	0.22^ef^	0.96^a^	0.73^b^	0.54^c^	0.47^cd^	0.33^def^	0.20^f^	0.36^de^	0.036	<0.001
C16:1	0.64	0.85	0.86	0.70	0.83	0.70	0.64	0.94	0.036	0.278
C17:1 (cis-10)	0.58^bc^	0.80^a^	0.75^a^	0.65^b^	0.61^b^	0.57^bc^	0.49^c^	0.56^bc^	0.017	<0.001
C17:1t	0.42^bc^	0.90^a^	0.54^b^	0.50^bc^	0.46^bc^	0.42^bc^	0.36^c^	0.40^bc^	0.027	<0.001
C18:1	0.09^de^	0.08^e^	0.09^bc^	0.09^bc^	0.09^de^	0.09^cd^	0.10^ab^	0.10^a^	0.001	<0.001
C18:1t	0.22^cd^	0.20^d^	0.28^ab^	0.28^abc^	0.24^bcd^	0.26^abc^	0.27^abc^	0.30^a^	0.007	0.002
C18:1n-9	0.12	0.13	0.14	0.14	0.12	0.13	0.13	0.17	0.005	0.090
C18:1n-9t	3.32^f^	7.06^c^	6.05^d^	6.55^cd^	8.53^b^	6.82^c^	5.08^e^	12.37^a^	0.332	<0.001
C19:1t (trans-7)	0.23^bc^	0.36^ab^	0.24^bc^	0.37^ab^	0.39^a^	0.16^c^	0.18^c^	0.28^abc^	0.019	0.001
C19:1t (trans-10)	0.45^b^	0.59^a^	0.51^ab^	0.51^ab^	0.51^ab^	0.49^ab^	0.44^b^	0.46^b^	0.012	0.042
C20:1	0.12^f^	0.24^d^	0.22^d^	0.24^d^	0.35^c^	0.44^b^	0.17^e^	0.52^a^	0.017	<0.001
C20:1t	0.20^e^	0.40^c^	0.37^c^	0.35^cd^	0.50^b^	0.53^b^	0.30^d^	0.70^a^	0.020	<0.001
C22:1t	0.11	0.17	0.13	0.13	0.13	0.10	0.17	0.10	0.009	0.334
C22:1n-9	0.33	0.44	0.35	0.31	0.32	0.40	0.28	0.30	0.015	0.138
C24:1	7.18^d^	8.30^bc^	9.05^a^	8.59^ab^	8.98^a^	8.07^bc^	6.73^d^	7.90^c^	0.116	<0.001
ΣMUFA	6.45^e^	11.49^c^	10.88^c^	10.70^c^	12.86^b^	10.59^c^	8.17^d^	16.59^a^	0.396	<0.001
C18:3n-3	0.49^e^	1.21^c^	0.76^d^	0.77^d^	1.65^b^	1.24^c^	0.73^d^	1.92^a^	0.062	<0.001
C20:3n-3	0.39^e^	0.47^cd^	0.42^de^	0.42^de^	0.71^b^	0.98^a^	0.49^c^	0.97^a^	0.031	<0.001
C20:5n-3	0.40^c^	0.49^a^	0.45^ab^	0.43^bc^	0.44^b^	0.43^b^	0.41^bc^	0.40^bc^	0.006	<0.001
C22:3n-3	8.83^bc^	9.66^bc^	11.32^bc^	27.75^a^	10.84^bc^	13.40^b^	5.92^c^	9.50^bc^	1.096	<0.001
C22:5n-3	1.06^e^	2.84^b^	2.08^c^	1.40^d^	2.71^b^	1.30^de^	1.94^c^	3.98^a^	0.121	<0.001
Σn-3 PUFA	11.10^bc^	14.51^bc^	14.85^bc^	30.43^a^	15.82^bc^	17.33^b^	9.31^c^	16.79^b^	1.064	<0.001
C18:2n-6	15.87^d^	42.47^b^	25.45^c^	24.20^c^	45.73^b^	44.09^b^	21.47^c^	56.92^a^	1.794	<0.001
C18:2n-6t	0.05^e^	0.15^a^	0.10^cd^	0.11^bc^	0.13^b^	0.09^cd^	0.08^d^	0.16^a^	0.005	<0.001
C18:3n-6	0.16^e^	0.84^c^	0.42^d^	0.42^d^	1.23^b^	0.84^c^	0.38^d^	1.51^a^	0.057	<0.001
C20:2n-6	0.23^f^	0.29^e^	0.06^g^	0.07^g^	0.57^c^	0.98^a^	0.36^d^	0.91^b^	0.044	<0.001
C20:3n-6	0.35^d^	0.48^c^	0.41^cd^	0.39^d^	0.75^b^	1.05^a^	0.48^c^	1.02^a^	0.037	<0.001
C20:4n-6	9.99^de^	11.41^c^	10.76^cd^	9.31^e^	15.21^b^	14.48^b^	11.44^c^	17.44^a^	0.366	<0.001
C22:2n-6	0.12	0.11	0.12	0.12	0.12	0.12	0.12	0.11	0.002	0.552
C22:4n-6	2.04	3.09	1.54	1.57	1.89	2.19	1.66	2.38	0.197	0.551
C22:5n-6	0.35^b^	0.36^b^	0.21^b^	0.21^b^	0.43^b^	0.39^b^	0.37^b^	0.80^a^	0.036	<0.001
Σn-6 PUFA	27.90^e^	59.12^c^	37.63^d^	35.95^d^	65.80^b^	64.24^bc^	36.20^d^	81.27^a^	2.369	<0.001
n-6/n-3 PUFA	2.63^c^	4.18^b^	2.61^c^	1.50^d^	4.16^b^	3.82^b^	3.97^b^	4.87^a^	0.156	<0.001
ΣPUFA	39.00^e^	73.64^bc^	50.63^de^	61.89^cd^	71.64^bc^	83.68^b^	44.35^e^	98.05^a^	2.901	<0.001
ΣUFA	45.45^f^	83.69^bc^	61.51^de^	72.59^cd^	84.50^bc^	94.48^b^	51.50^ef^	114.65^a^	3.212	<0.001
AI	0.75^a^	0.62^a^	0.64^a^	0.43^b^	0.61^a^	0.41^b^	0.61^a^	0.30^b^	0.026	<0.001
TI	1.04^ab^	0.80^bc^	0.84^abc^	0.52^d^	0.75^cd^	0.68^cd^	1.07^a^	0.59^cd^	0.037	<0.001
DHFA	63.09^g^	103.61^cd^	82.39^ef^	93.15^de^	111.85^bc^	123.33^b^	72.04^fg^	149.00^a^	3.910	<0.001
HH	0.49	0.94	0.78	2.56	1.12	1.49	0.92	2.57	0.230	0.152
HSFA	32.78^bc^	46.98^a^	35.55^b^	29.50^bc^	38.55^b^	31.81^bc^	26.18^c^	24.39^c^	1.282	<0.001

Data are expressed as means with their SEM (*n* = 8). ^a-f^ Different letters indicate differences (*p* < 0.05) between mean values tested by Duncan’s multiple range test. ΣSFA, sum of saturated fatty acids; ΣMUFA, sum of monounsaturated fatty acids; Σn-3 PUFA, sum of n-3 polyunsaturated fatty acids; Σn-6 PUFA, sum of n-6 polyunsaturated fatty acids; n-6/n-3 PUFA, ratio of Σn-6 PUFA to Σn-3 PUFA; ΣPUFA, sum of polyunsaturated fatty acids; ΣUFA, sum of unsaturated fatty acids; AI, atherogenic index; TI, thrombogenic index; DHFA, desirable hypocholesterolemic fatty acids; HH, hypocholesterolemic and hypercholesterolemic fatty acids ratio; HSFA, hypercholesterolemic saturated fatty acids. Control group, fed a basal diet; L group, basal diet supplemented with 0.50% *Leonuri herba*; LF group, basal diet supplemented with 0.25% *Ligustri lucidi fructus*; T group, basal diet supplemented with 0.25% *Taraxaci herba*; L-LF group, basal diet supplemented with 0.50% L + 0.25% LF; L-T group, basal diet supplemented with 0.50% L + 0.25% T; LF-T group, basal diet supplemented with 0.25% LF + 0.25% T; L-LF-T group, basal diet supplemented with 0.50% L + 0.25% LF + 0.25% T.

The effects of dietary CHUP on fatty acid composition in egg yolk on day 120 of the trial are summarized in Table 6. Compared with the control group, the L, LF, T, L-LF, and L-T groups had higher (*p*** **<** **0.05) concentrations of C16:1, C17:1 (cis-10), C17:1t, C19:1t (trans-10), C20:0, and C20:1t; the L and T groups had higher (*p*** **<** **0.05) SFAs (including C14:0, C17:0, C20:0, C21:0, and C24:0); the L, LF, T, L-LF, and L-T groups (particularly in the LF, T, and L-LF groups) had higher (*p*** **<** **0.05) MUFAs (including C16:1, C17:1 (cis-10), C17:1t, C19:1t (trans-10), and C20:1t); the LF-T group had higher (*p*** **<** **0.05) n-3 PUFAs, whereas the L-LF group had higher (*p*** **<** **0.05) concentrations of C18:2n-6, C18:3n-3, C18:3n-6, C20:2n-6, C20:3n-3, C20:3n-6, and C20:4n-6. The n-6 PUFAs, UFAs, and n-6/n-3 PUFA ratio in the L, T, and L-LF groups were higher (*p*** **<** **0.05), as well as the PUFAs in the L and L-LF groups, when compared with the control group. The AI and HSFA were decreased (*p*** **<** **0.05) in the L, LF, T, L-LF, L-T, and L-LF-T groups while increasing (*p*** **<** **0.05) in the LF-T group compared with the control group. Moreover, the DHFA was increased (*p*** **<** **0.05) in the L, T, and L-LF groups compared with the control group.

**Table 6.** Effects of Chinese herb ultrafine power (CHUP) on fatty acid composition in egg yolk (ng/mg of egg yolk, wet basis) of laying hens on day 120 of the trial.

Items	Dietary groups								SEM	*P*-values
	Control	L	LF	T	L-LF	L-T	LF-T	L-LF-T		
C6:0	1.34	1.59	1.50	2.10	1.68	1.80	0.87	1.92	0.108	0.136
C7:0	0.19^b^	0.28^b^	0.09^b^	0.61^a^	0.29^b^	0.12^b^	0.20^b^	0.16^b^	0.033	0.001
C11:0	0.32	0.33	0.30	0.20	0.21	0.17	0.10	0.16	0.024	0.145
C12:0	7.12^a^	2.32^b^	2.63^b^	1.95^b^	7.17^a^	7.28^a^	10.93^a^	9.10^a^	0.587	<0.001
C13:0	1.67	1.16	1.35	1.23	1.38	1.56	2.33	1.77	0.102	0.092
C14:0	1.17^d^	1.32^c^	0.97^e^	1.64^b^	2.57^a^	1.68^b^	0.95^e^	1.14^d^	0.066	<0.001
C15:0	0.36^b^	0.24^c^	0.17^d^	0.26^c^	0.61^a^	0.35^b^	0.26^c^	0.25^c^	0.017	<0.001
C16:0	39.13^b^	17.62^de^	15.18^e^	24.41^cd^	20.46^de^	24.62^cd^	59.23^a^	30.52^c^	1.949	<0.001
C17:0	0.16^d^	0.22^a^	0.21^ab^	0.20^bc^	0.19^c^	0.17^d^	0.14^e^	0.16^d^	0.004	<0.001
C18:0	24.09^b^	20.14^ef^	19.33^f^	23.41^bc^	28.66^a^	21.84^cd^	22.28^cd^	21.03^de^	0.392	<0.001
C20:0	3.37^de^	15.54^a^	13.12^ab^	11.07^bc^	11.57^b^	8.57^c^	1.60^e^	5.57^d^	0.665	<0.001
C21:0	19.75^d^	33.77^ab^	34.92^a^	30.97^ab^	29.01^c^	24.16^d^	19.10^d^	22.86^d^	0.927	<0.001
C22:0	1.96^b^	9.76^a^	3.58^b^	1.14^b^	1.52^b^	1.72^b^	3.74^b^	1.97^b^	0.451	<0.001
C23:0	14.32^c^	14.95^c^	21.91^a^	18.50^b^	17.45^b^	14.70^c^	14.33^c^	13.97^c^	0.404	<0.001
C24:0	20.99^c^	50.79^a^	42.49^a^	41.68^a^	32.33^b^	26.11^bc^	17.25^c^	26.18^bc^	1.766	<0.001
ΣSFA	133.40^bc^	166.22^a^	147.86^abc^	157.83^a^	151.92^abc^	134.81^bc^	153.25^ab^	131.78^c^	2.634	0.002
C15:1	0.12^c^	0.23^ab^	0.32^a^	0.36^a^	0.16^c^	0.24^ab^	0.17^c^	0.33^a^	0.019	0.002
C15:1t	0.26	0.30	0.20	0.39	0.39	0.29	0.11	0.43	0.030	0.116
C16:1	3.05^f^	3.80^de^	4.40^bc^	4.84^ab^	4.96^a^	4.04^cd^	2.15^g^	3.49^e^	0.124	<0.001
C17:1 (cis-10)	1.35^d^	2.41^a^	2.44^a^	2.24^a^	2.47^a^	1.92^b^	1.34^d^	1.65^c^	0.063	<0.001
C17:1t	1.05^d^	1.96^a^	1.96^a^	1.82^a^	1.94^a^	1.53^b^	1.00^d^	1.28^c^	0.054	<0.001
C18:1	0.09^bc^	0.08^c^	0.10^ab^	0.11^a^	0.09^bc^	0.09^bc^	0.10^c^	0.08^b^	0.002	0.001
C18:1t	0.22^b^	0.19^b^	0.25^b^	0.35^a^	0.25^b^	0.23^b^	0.37^a^	0.19^b^	0.011	<0.001
C18:1n-9	0.22^bcd^	0.19^cd^	0.12^d^	0.37^a^	0.31^ab^	0.23^bc^	0.13^cd^	0.30^ab^	0.015	<0.001
C18:1n-9t	5.05^ef^	6.14^d^	4.61^f^	8.38^b^	9.04^a^	6.86^c^	4.80^ef^	5.21^e^	0.215	<0.001
C19:1t (trans-7)	1.34^de^	3.89^a^	2.97^b^	2.61^b^	2.31^bc^	1.72^cd^	0.83^e^	1.58^cd^	0.143	<0.001
C19:1t (trans-10)	1.22^d^	2.51^a^	2.16^b^	1.92^bc^	2.07^b^	1.59^c^	0.96^d^	1.58^c^	0.075	<0.001
C20:1	0.09^d^	1.17^a^	0.65^b^	0.56^b^	0.53^bc^	0.36^bcd^	0.12^d^	0.25^cd^	0.053	<0.001
C20:1t	0.24^d^	1.27^a^	1.00^b^	0.88^bc^	0.92^bc^	0.67^c^	0.19^d^	0.41^d^	0.055	<0.001
C22:1t	0.12	0.13	0.17	0.15	0.13	0.14	0.16	0.16	0.008	0.795
C22:1n-9	0.35	0.32	0.35	0.36	0.31	0.36	0.32	0.32	0.011	0.685
C24:1	9.88^bc^	12.10^a^	10.49^b^	10.24^bc^	10.42^b^	9.41^c^	10.21^bc^	8.59^d^	0.153	<0.001
ΣMUFA	13.67^de^	22.08^b^	19.75^c^	23.41^ab^	24.46^a^	19.07^c^	12.01^e^	15.69^d^	0.598	<0.001
C18:3n-3	0.90^b^	0.94^b^	0.65^d^	0.96^b^	1.04^a^	0.75^c^	0.54^e^	0.75^c^	0.021	<0.001
C20:3n-3	0.62^b^	0.58^bc^	0.40^f^	0.50^e^	0.80^a^	0.51^de^	0.54^cd^	0.49^e^	0.015	<0.001
C20:5n-3	0.44^ab^	0.45^a^	0.41^abc^	0.38^bc^	0.45^a^	0.36^c^	0.47^a^	0.38^bc^	0.009	0.002
C22:3n-3	16.63^b^	8.43^d^	11.58^cd^	8.89^d^	12.96^bc^	11.06^cd^	21.06^a^	13.69^bc^	0.666	<0.001
C22:5n-3	1.87^c^	0.90^e^	0.83^e^	3.10^a^	2.73^b^	1.36^d^	1.53^d^	1.78^c^	0.099	<0.001
Σn-3 PUFA	20.47^b^	11.35^d^	17.00^cd^	13.86^cd^	20.78^b^	15.91^cd^	17.10^a^	24.15^bc^	0.669	<0.001
C18:2n-6	23.41^c^	38.73^a^	24.82^c^	32.38^b^	38.70^a^	23.67^c^	19.95^d^	22.57^cd^	0.945	<0.001
C18:2n-6t	0.10^a^	0.06^bc^	0.04^c^	0.07^b^	0.07^ab^	0.07^ab^	0.08^ab^	0.07^b^	0.004	0.002
C18:3n-6	0.55^c^	0.59^b^	0.30^e^	0.60^b^	0.68^a^	0.47^d^	0.22^f^	0.43^d^	0.020	<0.001
C20:2n-6	0.53^b^	0.49^b^	0.17^de^	0.16^e^	0.97^a^	0.27^cd^	0.33^c^	0.56^b^	0.033	<0.001
C20:3n-6	0.60^b^	0.55^b^	0.37^d^	0.46^c^	0.84^a^	0.49^c^	0.59^b^	0.45^c^	0.018	<0.001
C20:4n-6	10.58^c^	7.75^e^	7.51^e^	9.45^d^	12.61^b^	7.47^e^	14.48^a^	9.19^d^	0.311	<0.001
C22:2n-6	0.13	0.14	0.14	0.14	0.14	0.14	0.13	0.14	0.002	0.563
C22:4n-6	1.66^b^	6.87^a^	2.38^b^	3.45^b^	1.55^b^	2.38^b^	1.16^b^	2.95^b^	0.357	<0.001
C22:5n-6	0.25	0.26	0.20	0.41	0.58	0.22	0.38	0.43	0.035	0.079
Σn-6 PUFA	37.74^c^	55.33^a^	35.58^c^	47.10^b^	55.94^a^	34.83^c^	35.35^c^	36.68^c^	1.215	<0.001
n-6/n-3 PUFA	1.91^ef^	5.14^a^	2.77^cd^	3.53^b^	3.22^bc^	2.46^de^	1.48^f^	2.17^def^	0.164	<0.001
ΣPUFA	56.77^c^	66.58^b^	47.71^d^	60.89^bc^	74.53^a^	47.14^d^	59.49^bc^	53.78^cd^	1.389	<0.001
ΣUFA	70.14^c^	88.66^b^	67.46^c^	84.30^b^	99.18^a^	66.21^c^	71.50^c^	69.47^c^	1.711	<0.001
AI	0.63^b^	0.26^d^	0.28^d^	0.34^d^	0.32^d^	0.48^c^	0.89^a^	0.46^c^	0.030	<0.001
TI	0.75^abc^	0.55^bc^	0.50^c^	0.65^abc^	0.55^bc^	0.87^a^	0.82^ab^	0.64^abc^	0.033	0.025
DHFA	93.83^c^	108.80^b^	86.78^c^	107.70^b^	128.12^a^	88.05^c^	93.78^c^	90.49^c^	1.978	<0.001
HH	3.06	2.09	1.82	1.45	1.75	0.96	0.35	3.69	0.464	0.720
HSFA	40.29^b^	18.94^de^	16.16^e^	26.05^cd^	23.00^de^	26.30^cd^	60.18^a^	31.65^c^	1.928	<0.001

Data are expressed as means with their SEM (*n* = 8). ^a-f^ Different letters indicate differences (*p* < 0.05) between mean values tested by Duncan’s multiple range test. ΣSFA, sum of saturated fatty acids; ΣMUFA, sum of monounsaturated fatty acids; Σn-3 PUFA, sum of n-3 polyunsaturated fatty acids; Σn-6 PUFA, sum of n-6 polyunsaturated fatty acids; n-6/n-3 PUFA, ratio of Σn-6 PUFA to Σn-3 PUFA; ΣPUFA, sum of polyunsaturated fatty acids; ΣUFA, sum of unsaturated fatty acids; AI, atherogenic index; TI, thrombogenic index; DHFA, desirable hypocholesterolemic fatty acids; HH, hypocholesterolemic and hypercholesterolemic fatty acids ratio; HSFA, hypercholesterolemic saturated fatty acids. Control group, fed a basal diet; L group, basal diet supplemented with 0.50% *Leonuri herba*; LF group, basal diet supplemented with 0.25% *Ligustri lucidi fructus*; T group, basal diet supplemented with 0.25% *Taraxaci herba*; L-LF group, basal diet supplemented with 0.50% L + 0.25% LF; L-T group, basal diet supplemented with 0.50% L + 0.25% T; LF-T group, basal diet supplemented with 0.25% LF + 0.25% T; L-LF-T group, basal diet supplemented with 0.50% L + 0.25% LF + 0.25% T.

### Effects of CHUP on amino acid composition in egg albumen

**Figure 3.**
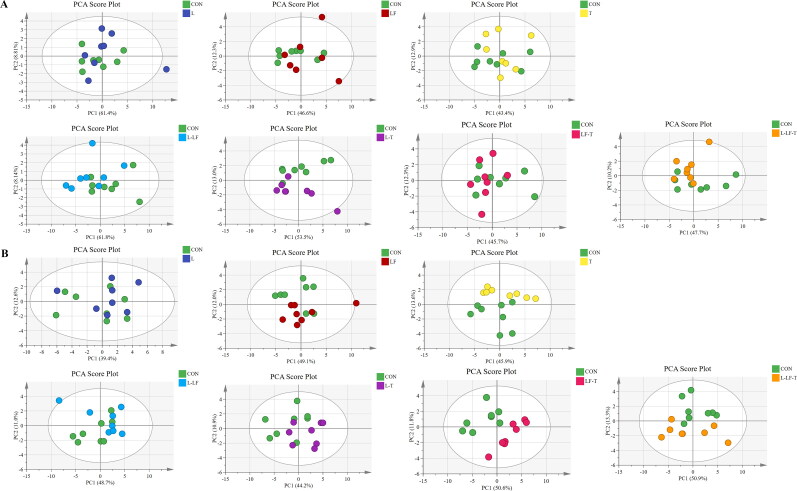
Principal component analysis (PCA) scores plots. Amino acid targeted metabolomics of the albumen on days 60 (A) and 120 (B) of the trial. Control group (CON), fed a basal diet; L group, basal diet supplemented with 0.50% *Leonuri herba*; LF group, basal diet supplemented with 0.25% *Ligustri lucidi fructus*; T group, basal diet supplemented with 0.25% *Taraxaci herba*; L-LF group, basal diet supplemented with 0.50% L + 0.25% LF; L-T group, basal diet supplemented with 0.50% L + 0.25% T; LF-T group, basal diet supplemented with 0.25% LF + 0.25% T; L-LF-T group, basal diet supplemented with 0.50% L + 0.25% LF + 0.25% T.

**Figure 4.**
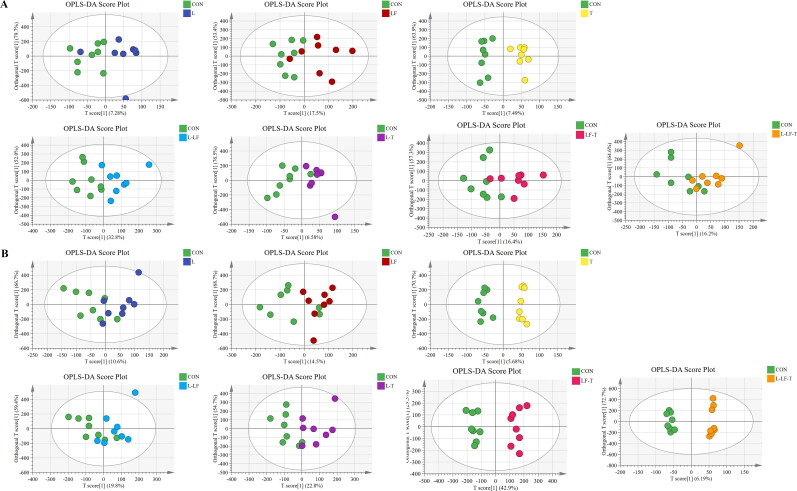
Orthogonal partial least squares-discriminant analysis (OPLS-DA) scores plots of amino acid on days 60 (A) and 120 (B) of the trial. Control group (CON), fed a basal diet; L group, basal diet supplemented with 0.50% *Leonuri herba*; LF group, basal diet supplemented with 0.25% *Ligustri lucidi fructus*; T group, basal diet supplemented with 0.25% *Taraxaci herba*; L-LF group, basal diet supplemented with 0.50% L + 0.25% LF; L-T group, basal diet supplemented with 0.50% L + 0.25% T; LF-T group, basal diet supplemented with 0.25% LF + 0.25% T; L-LF-T group, basal diet supplemented with 0.50% L + 0.25% LF + 0.25% T.

The PCA and OPLS-DA analysis results of amino acid are shown in Figures 3 and 4. OPLS-DA scores plots (Figure 4) showed obvious separations between the T, L-LF, and control groups on day 60 of the trial. However, on day 120 of the trial, the PCA (Figure 3) and OPLS-DA scores plots displayed distinct separations between the T, LF-T, L-LF-T, and control groups. The PCA and OPLS-DA analysis for amino acid showed that the treatment and control groups were not distinctly separated as those were showed for fatty acid, indicating that dietary CHUP had better impacts on the fatty acid composition in egg yolks than amino acid.

Amino acid composition in egg albumen on day 60 of the trial is presented in Table 7. The level of lysine was increased (*p*** **<** **0.05) in the L group, as well as the proline in the T group, whereas the levels of alanine, arginine, glutamic acid, histidine, isoleucine, phenylalanine, proline, the sum of ARAA, BAA, BCAA, EAA, and TAA were decreased (*p*** **<** **0.05) in the L-LF group, when compared with the control group. Additionally, the levels of alanine, arginine, glutamic acid, histidine, phenylalanine, proline, EAA, and TAA were increased (*p*** **<** **0.05) in the L, LF, and T groups, as well as histidine, isoleucine, leucine, methionine, phenylalanine, proline, ARAA, and BCAA in the L-T group, when compared with the L-LF group. The LF and T groups had higher (*p*** **<** **0.05) levels of alanine, histidine, isoleucine, BCAA, and TAA, whereas the L group had higher (*p*** **<** **0.05) levels of histidine, lysine, and BAA, when compared with the LF-T and L-LF-T groups. Interestingly, the LF group had higher (*p*** **<** **0.05) levels of alanine, EAA, and TAA compared with the L-LF, L-T, LF-T, and L-LF-T groups.

**Table 7.** Effects of Chinese herb ultrafine power (CHUP) on amino acid composition in egg albumen (µg/mL of egg albumen, wet basis) of laying hens on day 60 of the trial.

Items	Dietary groups								SEM	*P*-values
	Control	L	LF	T	L-LF	L-T	LF-T	L-LF-T		
Ala	1.06^abc^	1.11^abc^	1.21^a^	1.19^ab^	0.78^d^	0.98^bcd^	0.96^cd^	0.93^cd^	0.029	0.001
Arg	2.83^a^	2.91^a^	2.92^a^	2.84^a^	1.94^b^	2.41^ab^	2.61^a^	2.50^ab^	0.078	0.013
Asp	1.13	1.23	1.29	1.44	0.96	1.04	1.26	0.77	0.060	0.168
Cys	0.62	0.99	0.85	0.97	1.15	1.06	0.97	1.20	0.062	0.378
Glu	6.08^a^	6.25^a^	6.24^a^	6.29^a^	4.91^b^	5.18^ab^	5.62^ab^	4.68^b^	0.145	0.007
Gly	5.09	5.66	3.82	2.97	2.55	3.04	2.90	4.12	0.319	0.133
His	1.93^ab^	2.21^a^	2.25^a^	2.25^a^	1.46^c^	1.96^ab^	1.77^bc^	1.71^bc^	0.056	<0.001
Ile	16.58^ab^	14.61^abc^	17.67^a^	18.27^a^	12.69^c^	16.58^ab^	13.50^bc^	13.38^bc^	0.474	0.007
Leu	15.45^abcd^	15.09^abcd^	17.76^a^	16.95^ab^	12.50^d^	16.08^abc^	14.21^bcd^	13.47^cd^	0.393	0.007
Lys	3.34^b^	4.54^a^	3.07^bc^	2.63^bc^	2.84^bc^	2.16^c^	3.10^bc^	2.97^bc^	0.132	<0.001
Met	1.46^abc^	1.45^abc^	1.63^ab^	1.64^a^	1.19^c^	1.59^ab^	1.28^c^	1.33^bc^	0.038	0.008
Phe	35.05^ab^	33.73^ab^	38.98^a^	35.49^ab^	26.53^c^	34.01^ab^	30.16^bc^	31.97^bc^	0.836	0.008
Pro	1.31^bc^	1.27^bc^	1.64^ab^	1.98^a^	0.88^d^	1.36^bc^	1.36^bc^	1.11^cd^	0.057	<0.001
Ser	0.14	0.14	0.15	0.14	0.14	0.13	0.13	0.14	0.002	0.518
Thr	0.69	0.86	0.80	0.78	0.59	0.69	0.78	0.66	0.025	0.129
Try	1.94	1.91	1.98	1.84	1.55	1.86	1.76	1.70	0.038	0.074
Tyr	38.87	36.22	47.51	43.04	33.13	42.46	40.07	36.69	1.161	0.050
Val	1.57^abc^	1.81^ab^	2.01^a^	1.88^ab^	1.35^c^	1.79^abc^	1.65^abc^	1.50^bc^	0.054	0.038
AAA	7.21^ab^	7.35^ab^	7.55^a^	7.74^a^	5.87^bc^	6.22^abc^	6.88^abc^	5.45^c^	0.189	0.014
ARAA	73.92^ab^	71.38^abc^	86.49^a^	76.52^ab^	58.39^c^	76.46^ab^	70.23^bc^	68.66^bc^	1.885	0.018
BAA	8.09^abc^	9.30^a^	8.44^ab^	7.73^abcd^	6.24^d^	6.35^cd^	7.48^bcd^	7.08^bcd^	0.226	0.006
BCAA	33.60^ab^	31.51^abc^	37.44^a^	36.33^a^	26.54^c^	34.44^ab^	29.35^bc^	28.34^bc^	0.868	0.006
EAA	76.08^abc^	74.70^abc^	82.98^a^	78.52^ab^	57.66^d^	67.03^bcd^	64.94^cd^	66.98^bcd^	1.710	0.001
SAA	2.08	2.44	2.39	2.60	2.37	2.64	2.27	2.53	0.077	0.662
TAA	129.96^abc^	133.66^abc^	147.83^a^	139.19^ab^	101.43^d^	120.13^bcd^	118.27^cd^	116.50^cd^	2.847	<0.001

Data are expressed as means with their SEM (*n* = 8). ^a-d^ Different letters indicate differences (*p* < 0.05) between mean values tested by Duncan’s multiple range test. Ala, alanine; Arg, arginine; Asp, aspartic acid; Cys, cystine; Glu, glutamic acid; Gly, glycine; His, histidine; Ile, isoleucine; Leu, leucine; Lys, lysine; Met, methionine; Phe, phenylalanine; Pro, proline; Ser, serine; Thr, threonine; Try, tryptophan; Tyr, tyrosine; Val, valine; AAA, acidic amino acid; ARAA, aromatic amino acid; BAA, basic amino acid; BCAA, Branched-chain amino acid; EAA, the sum of essential amino acid; SAA, sulphur-containing amino acid; TAA, total amino acid. Control group, fed a basal diet; L group, basal diet supplemented with 0.50% *Leonuri herba*; LF group, basal diet supplemented with 0.25% *Ligustri lucidi fructus*; T group, basal diet supplemented with 0.25% *Taraxaci herba*; L-LF group, basal diet supplemented with 0.50% L + 0.25% LF; L-T group, basal diet supplemented with 0.50% L + 0.25% T; LF-T group, basal diet supplemented with 0.25% LF + 0.25% T; L-LF-T group, basal diet supplemented with 0.50% L + 0.25% LF + 0.25% T.

Amino acid composition in egg albumen on day 120 of the trial is presented in Table 8. The lysine level was increased (*p*** **<** **0.05) in the LF-T and L-LF-T groups, while alanine, histidine, leucine, methionine, phenylalanine, proline, BCAA, EAA, and TAA levels were decreased (*p*** **<** **0.05) in the L-LF-T group, when compared with the control group. In addition, the levels of leucine, phenylalanine, proline, BCAA, and EAA were higher (*p*** **<** **0.05) in the L, LF, and T groups, as well as leucine and proline in the L-LF, L-T, and LF-T groups, when compared with the L-LF-T group.

**Table 8.** Effects of Chinese herb ultrafine power (CHUP) on amino acid composition in egg albumen (µg/mL of egg albumen, wet basis) of laying hens on day 120 of the trial.

Items	Dietary groups								SEM	*P*-values
	Control	L	LF	T	L-LF	L-T	LF-T	L-LF-T		
Ala	1.47^a^	1.45^a^	1.52^a^	1.17^b^	1.17^b^	1.14^b^	1.36^ab^	1.15^b^	0.035	0.004
Arg	3.65	3.68	4.33	3.89	3.47	3.22	3.85	3.27	0.099	0.133
Asp	1.69	1.40	1.69	1.75	1.58	1.37	1.14	1.27	0.073	0.344
Cys	1.43	1.65	1.30	1.16	1.77	0.95	1.21	1.34	0.069	0.076
Glu	6.89	7.10	7.76	7.10	6.62	6.79	7.32	6.46	0.162	0.624
Gly	5.24	3.95	6.35	5.62	4.20	3.45	5.74	4.41	0.279	0.106
His	2.97^ab^	2.64^abc^	3.12^a^	2.75^abc^	2.69^abc^	2.46^bc^	2.72^abc^	2.15^c^	0.074	0.046
Ile	20.97	21.04	23.04	20.94	18.44	20.64	19.94	15.21	0.619	0.070
Leu	23.88^a^	21.13^a^	22.98^a^	20.99^a^	20.23^a^	20.98^a^	20.73^a^	16.07^b^	0.549	0.022
Lys	2.39^c^	2.67^bc^	3.07^abc^	3.20^abc^	2.74^bc^	2.59^bc^	3.33^ab^	3.61^a^	0.100	0.022
Met	2.18^a^	1.76^b^	2.20^a^	1.88^ab^	1.84^ab^	1.83^ab^	1.83^ab^	1.58^b^	0.049	0.020
Phe	52.94^a^	47.19^ab^	48.00^ab^	47.26^ab^	45.85^ab^	41.57^bc^	44.75^ab^	34.16^c^	1.318	0.022
Pro	2.42^a^	2.20^abc^	2.37^ab^	1.80^bc^	1.72^c^	1.76^c^	1.98^abc^	1.18^d^	0.078	<0.001
Ser	0.12	0.13	0.12	0.13	0.13	0.13	0.14	0.14	0.002	0.277
Thr	1.00	1.13	1.02	1.00	1.00	0.78	0.99	0.74	0.034	0.095
Try	2.41	2.54	2.43	2.46	2.36	2.31	2.37	1.96	0.057	0.298
Tyr	54.52	49.51	53.64	51.65	48.12	48.81	50.31	39.53	1.369	0.175
Val	2.55	2.37	2.51	2.15	2.14	2.05	2.27	1.83	0.066	0.104
AAA	8.66	8.51	9.59	9.11	8.12	8.16	8.26	7.73	0.184	0.242
ARAA	107.46	95.01	101.64	98.91	93.97	90.38	95.06	73.70	2.595	0.059
BAA	9.01	9.00	10.68	9.85	8.90	8.24	9.89	9.03	0.234	0.274
BCAA	46.28^ab^	44.55^ab^	50.64^a^	44.08^ab^	40.07^bc^	43.67^ab^	42.94^ab^	33.12^c^	1.144	0.010
EAA	101.03^a^	95.79^a^	107.61^a^	99.86^a^	92.34^ab^	92.74^ab^	89.99^ab^	75.37^b^	2.284	0.038
SAA	3.61	3.41	3.58	3.04	3.61	2.77	2.95	2.92	0.099	0.133
TAA	181.32^ab^	168.79^abc^	193.29^a^	183.64^ab^	159.48^abc^	159.51^abc^	154.96^bc^	135.38^c^	4.198	0.015

Data are expressed as means with their SEM (*n* = 8). ^a-c^ Different letters indicate differences (*p* < 0.05) between mean values tested by Duncan’s multiple range test. Ala, alanine; Arg, arginine; Asp, aspartic acid; Cys, cystine; Glu, glutamic acid; Gly, glycine; His, histidine; Ile, isoleucine; Leu, leucine; Lys, lysine; Met, methionine; Phe, phenylalanine; Pro, proline; Ser, serine; Thr, threonine; Try, tryptophan; Tyr, tyrosine; Val, valine; AAA, acidic amino acid; ARAA, aromatic amino acid; BAA, basic amino acid; BCAA, Branched-chain amino acid; EAA, the sum of essential amino acid; SAA, sulphur-containing amino acid; TAA, total amino acid. Control group, fed a basal diet; L group, basal diet supplemented with 0.50% *Leonuri herba*; LF group, basal diet supplemented with 0.25% *Ligustri lucidi fructus*; T group, basal diet supplemented with 0.25% *Taraxaci herba*; L-LF group, basal diet supplemented with 0.50% L + 0.25% LF; L-T group, basal diet supplemented with 0.50% L + 0.25% T; LF-T group, basal diet supplemented with 0.25% LF + 0.25% T; L-LF-T group, basal diet supplemented with 0.50% L + 0.25% LF + 0.25% T.

### Correlation between egg quality, plasma reproductive hormones, TCEY, and fatty acid and amino acid in laying hens

**Figure 5.**
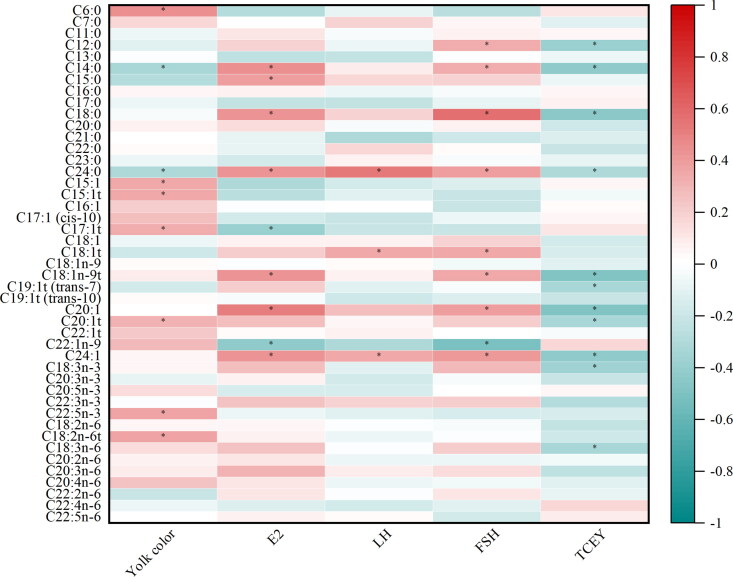
Spearman’s correlation analysis between yolk color, plasma reproductive hormones, TCEY, and the content of fatty acid. Red represents a significant positive correlation, and green represents a significant negative correlation. * *p* ≤ 0.05. FSH, follicle-stimulating hormone; LH, luteinizing hormone; E2, estradiol; TCEY, the total cholesterol in egg yolk.

Spearman’s correlation analysis was performed to explore the content of fatty acid in egg yolk associated with yolk color, plasma reproductive hormones, and TCEY (Figure 5). The yolk color was positively correlated (*p*** **<** **0.05) with C6:0, C15:1, C15:1t, C17:1t, C18:2n-6t, C20:1t, and C22:5n-3, whereas it was negatively correlated (*p*** **<** **0.05) with C14:0 and C24:0. The TCEY was negatively correlated (*p*** **<** **0.05) with C12:0, C14:0, C18:0, C18:1n-9t, C18:3n-3, C18:3n-6, C19:1t (trans-7), C20:1, C20:1t, C24:0, and C24:1. The FSH, LH, and E2 were positively correlated (*p*** **<** **0.05) with C24:0 and C24:1. The FSH and E2 were positively correlated (*p*** **<** **0.05) with C14:0, C18:0, C18:1n-9t, and C20:1, whereas those were negatively correlated (*p*** **<** **0.05) with C22:1n-9 level. Moreover, the FSH and LH were positively correlated (*p*** **<** **0.05) with C18:1t, and FSH was positively correlated (*p*** **<** **0.05) with C12:0. The E2 was positively correlated (*p*** **<** **0.05) with C15:0, while it was negatively correlated (*p*** **<** **0.05) with C17:1t.

**Figure 6.**
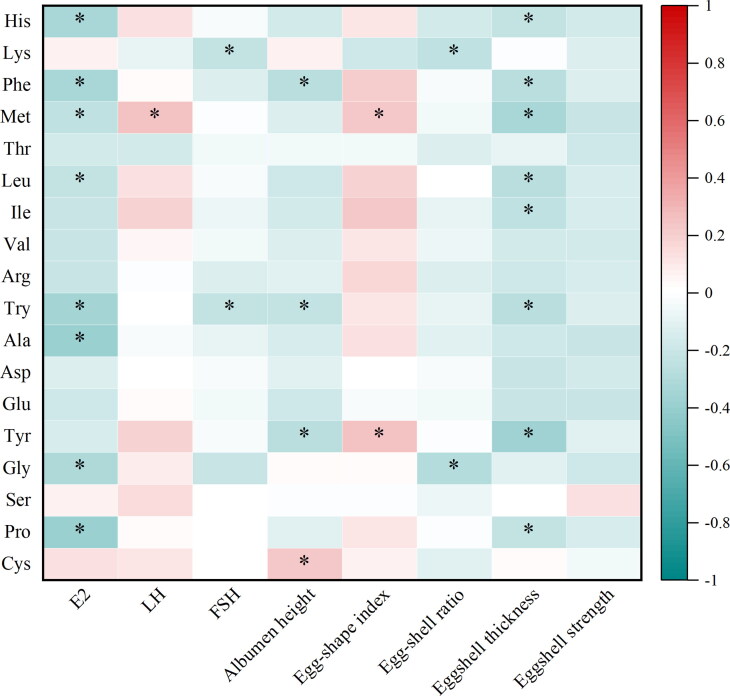
Spearman’s correlation analysis between egg quality, plasma reproductive hormones, and the content of amino acid. Red represents a significant positive correlation, and green represents a significant negative correlation. * *p* ≤ 0.05. His, histidine; Lys, lysine; Phe, phenylalanine; Met, methionine; Thr, threonine; Leu, leucine; Ile, isoleucine; Val, valine; Arg, arginine; Try, tryptophan; Ala, alanine; Asp, aspartic acid; Glu, glutamic acid; Tyr, tyrosine; Gly, glycine; Ser, serine; Pro, proline; Cys, cystine; FSH, follicle-stimulating hormone; LH, luteinizing hormone; E2, estradiol.

Spearman’s correlations between egg quality, plasma reproductive hormones, and amino acid in albumen are shown in Figure 6. The E2 and eggshell thickness were negatively correlated (*p*** **<** **0.05) with histidine, phenylalanine, methionine, leucine, tryptophan, and proline. The E2 was negatively correlated (*p*** **<** **0.05) with alanine and glycine, while eggshell thickness was negatively correlated (*p*** **<** **0.05) with isoleucine and tyrosine. The FSH was negatively correlated (*p*** **<** **0.05) with lysine and tryptophan, while LH was positively correlated (*p*** **<** **0.05) with methionine. The albumen height was negatively correlated (*p*** **<** **0.05) with phenylalanine, tryptophan, and tyrosine, whereas it was positively correlated (*p*** **<** **0.05) with cystine. Moreover, the eggshape index was ­positively correlated (*p*** **<** **0.05) with methionine and tyrosine, while eggshell ratio was negatively correlated (*p*** **<** **0.05) with lysine and glycine.

## Discussion

Eggs play critical roles as nutraceuticals in improving global health. During the late laying period, laying hens suffer a variety of physical overloads and inadequate nutritional intake, resulting in a significant decline in laying performance and egg quality. Therefore, the present study evaluated the effects of dietary CHUP supplementation on nutritional values and egg quality in laying hens. The results show that dietary CHUP enhanced quality and nutritional values of eggs in laying hens during the late laying stage.

Feed is one of the most important and main expenditures related to egg production (Clark et al. 2019). Feed intake is one of the most crucial productivity factors in flocks of laying hens (Akter et al. 2018). The present study indicated that dietary L-LF-T supplementation only reduced the ADFI during 1 − 30 days of the trial, which could be attributed to poor palatability of diets due to the addition of CHUP (Zhai et al. 2013). Other researchers reported that dietary supplementation of 0.5, 1.0, and 1.5% T (Cao et al. 2023) and 1% and 2% LF (Chen et al. 2020) could enhance egg production rate and reduce FCR in laying hens. However, no changes were observed in egg production, egg weight, and FCR in the present study, which may be related to inadequate amounts of CHUP additives, indicating that dietary CHUP had no effects on the absorption and utilization of feed nutrients in laying hens. These findings suggest that dietary L-LF-T supplementation might have poor palatability; however, it does not affect the nutrient efficiency of laying hens.

The edible quality and commercial value of eggs are directly affected by the external and intrinsic quality standards of eggs. External quality parameters of eggs include eggshell strength, eggshell thickness, egg weight (Dilawar et al. 2021), etc. Eggshell quality is one of the most important aspects affecting the poultry business, and it has an economic impact on egg output and hatchability (Ketta and Tůmová 2016). Eggshell strength is one of the most important markers of egg quality since flaws in eggshells result in a significant commercial loss (Stefanello et al. 2014). The percentage of eggs destroyed during transport and handling is related to eggshell thickness (Anderson et al. 2004). In the present study, both compound formula and single Chinese herb may improve the eggshell quality and commercial profit in laying hens by increasing the strength and thickness of eggshells. In addition, we found that several indicators, such as eggshell ratio, eggshell thickness, and yolk color, were affected only in the middle of the trial, but not in the last month. The reason might be explained by the fact of the poor health of the laying hens due to long-term egg production and inadequate amounts of CHUP supplementation.

Recently, it has been reported that the strength and thickness of eggshells were associated with the interaction of genotype, age, Ca (Ketta and Tůmová 2016), and reproductive hormones (Liu et al. 2013). Moreover, Ca plays an important role in regulating the reproductive hormones and ovary growth (Attia et al. 2020). Zhang et al. (2011) observed that the L could regulate serum hormone levels in older rats, and the Ca content was higher in T. In addition, dietary L, LF, T, and *Salvia miltiorrhiza Bge.* could up-regulate mRNA expressions of genes associated with eggshell formation (Han et al. 2023), suggesting that CHUP may improve the strength and thickness of eggshells by effecting reproductive hormones or Ca in laying hens. However, dietary L-LF, L-T, and LF-T supplementation decreased eggshell thickness on day 120 of the trial, which warrants further study. These findings suggest that dietary CHUP could improve eggshell quality by enhancing the strength and thickness of eggshells.

The external environment affects laying performance in hens, which is controlled by endocrine and genetic factors (Du et al. 2020). The FSH is the primary hormone involved in the formation and maturation of tiny follicles, especially F6 to F3 follicles and SYFs (Hernandez and Bahr 2003). In addition, E2 is critical to the preservation of female reproductive function in the ovary (Oride et al. 2023). The FSH and E2 could promote the expression of gonadotropin-releasing hormone (GnRH) (Thompson and Kaiser 2014), and the increase of GnRH can increase the reactivity of follicles to FSH and LH, and thus eventually facilitate the maturity of follicles (Xu et al. 1995). In the present study, dietary LF, T, L-LF, and L-T supplementation increased the plasma FSH level; moreover, dietary L-T, LF-T, and L-LF-T supplementation increased the plasma E2 level in laying hens on day 60 of the trial. These findings suggest that dietary CHUP supplementation contributed to improve the growth of follicles and ovulation by increasing the plasma FSH and E2 levels.

Fatty acids are essential for normal functions of all organisms (Czumaj and Śledziński 2020), and have direct impacts on the development and occurrence of metabolic diseases (Jankowska et al. 2010). UFAs are beneficial against oxidative stress, inflammation, cerebrovascular disorders, cancer, and osteoporosis (Liu et al. 2023). In the present study, dietary CHUP supplementation increased the MUFAs, n-6 PUFAs, and UFAs, while dietary L-LF-T had higher impacts on the concentrations of α-linolenic acid, eicosatrienoic acid, docosapentaenoic acid, elaidic acid, linoleic acid, and γ-linolenic acid in egg yolk on day 60 of the trial. However, dietary L-LF had greater benefits for egg yolk on day 120 of the trial. Since L and T are heat-clearing herbs with bitter and cold-cool nature (Yang et al. 2016), the L-LF-T is also a formula with cold properties. The long-term application of L-LF-T may lead to coldness in the spleen and stomach and then cause side effects on the health of laying hens. However, LF is a kind of Yin-tonifying herb (Hu et al. 2014), which can regulate the cold of L, so the L-LF is more effective for laying hens on day 120 of the trial. These findings suggest that dietary L-LF and L-LF-T supplementation might increase the UFAs concentration in egg yolk during the short- and long-term, respectively, and have protective effects against neurologic and cardiovascular diseases (CVDs). Since little information exists on the pharmacodynamic mechanisms of compound formula, it is postulated that combining LF with either L or T may increase its tonic action.

The current diet guidelines for humans recommend to decrease SFA-rich meals and increase UFA intake for CVDs prevention (Hoenselaar 2011). It is worth noting that dietary L and T supplementation increased the SFA concentration in egg yolk, which may contribute to an elevated atherosclerotic cardiovascular disease risk in humans (Maki et al. 2021). Therefore, dietary CHUP supplementation may have favorable effects on lipid profiles and risk factors for CVDs, allowing consumers to avoid obesity and hypercholesterolemia. Hypercholesterolemia is one of the primary causes of the development and progression of CVDs (Ortega et al. 2006). A previous study reported that AI might be useful in predicting the early detection of atherosclerotic CVDs and coronary artery disease (CAD) incidence (Mahdavi-Roshan et al. 2022). In the present study, dietary CHUP supplementation decreased the AI, and dietary T, L-LF, L-T, and L-LF-T supplementation decreased the total cholesterol level in egg yolk, which might be attributed to the fact that T and LF had triglyceride accumulation inhibitory (Zhang et al. 2013) and hypolipidemic (Liu et al. 2014) effects in HepG2 cells. These findings suggest that consumers can improve their health by choosing eggs selectively, not only to increase their intake of UFA, but also to prevent the occurrence of CVDs and CAD.

The increase in amino acid content of eggs leads to an enhancement in the nutritional values and flavors of eggs (Goto et al. 2021a). Although EAA should be consumed on a regular basis, they have an unpleasant bitter taste for humans. For instance, histidine, lysine (Camacho et al. 2023), leucine, methionine, (Akitomi et al. 2013), and phenylalanine (Jioe et al. 2023) indicate bitter taste. In addition, the content of amino acid could be altered by genetic and dietary factors (Goto et al. 2021b). In the present study, dietary L-LF supplementation on day 60 and dietary L-LF-T on day 120 of the trial reduced the content of histidine, lysine, leucine, methionine, and phenylalanine in egg albumen. Therefore, these findings suggest that dietary L-LF and L-LF-T supplementation can reduce the content of bitter amino acids in eggs at different times and improve the taste of eggs.

In the phenotypic correlation analysis, there were positive correlations between UFA and yolk color in this study. Panaite et al. (2019) reported that yolk color was positively correlated with the content of carotenoids in yolk, suggesting some associations between UFA and carotenoids. In the present study, UFAs, especially C18:1n-9t, C19:1t (trans-7), C20:1, C20:1t, and C24:1 were negatively correlated with the total cholesterol in egg yolk, and dietary CHUP supplementation could increase the content of UFAs and decrease the cholesterol content. Moreover, it has been reported that the correlation between omega-3 fatty acids consumption and plasma cholesterol was negative in muscovy ducklings (El-Deek et al. 1997). These results suggest that there are some associations between carotenoids, cholesterol, and UFAs. In addition, there was a negative correlation between the E2, eggshell thickness, and bitter amino acids, which may be partly responsible for decreasing the content of bitter amino acids in egg albumen by dietary L-LF and L-LF-T supplementation. However, further studies are needed to elucidate the possible causes.

## Conclusion

In summary, dietary CHUP supplementation could improve eggshell quality by increasing the strength and thickness of eggshells and enhancing the reproductive hormone levels of laying hens. Moreover, dietary CHUP supplementation during the late laying period could also increase the UFAs in egg yolk and improve the egg’s taste by reducing the content of bitter amino acids in egg albumen. These results reveal that dietary CHUP supplementation during the late laying period has positive effects on eggshell quality and the egg’s taste and enhances the UFAs in eggs. Among different CHUP, the L-LF and L-LF-T had better impacts and could be a potential and practical strategy to contribute to the production of laying hens. However, the application of dietary CHUP in different breeds of laying hens or farming conditions is still unknown and warrants further research. Nevertheless, these findings will provide a theoretical basis for the application of dietary CHUP in laying hens, and dietary CHUP could be a potential feed additive in poultry production.

## Data Availability

The data presented in this study are deposited in online repositories and accession number can be found at https://www.scidb.cn/en/anonymous/VlpmSUZm.
